# From Conquests to Epidemics in 18th-Century South America: A Reflection on Social Resilience and Reconstruction: Review of the Literature

**DOI:** 10.3390/epidemiologia5040049

**Published:** 2024-11-22

**Authors:** Jorge Hugo Villafañe

**Affiliations:** 1 Departamento de Historia y Filosofía, Universidad de Alcalá, 28801 Alcala de Henares, Spain; mail@villafane.it; 2 Faculty of Sport Sciences, Universidad Europea de Madrid, 28670 Villaviciosa de Odón, Spain

**Keywords:** resilience, health crises, South America

## Abstract

Background/Objectives: This narrative review examines resilience and social reconstruction strategies implemented during the 1742–1743 plague along the Royal Road between Buenos Aires and Lima. The study explores how colonial authorities managed the epidemic and its long-term effects, providing insights into historical crisis management and public health governance. Methods: A systematic analysis of primary and secondary historical records was conducted to identify public health measures, such as quarantines, hospital construction, and administrative reforms. Sources were retrieved from archives and databases, focusing on resilience strategies and institutional responses to the epidemic. Results: The findings highlight key public health interventions designed to contain the epidemic and mitigate its impacts. These included the establishment of quarantines, the construction of temporary hospitals, and administrative adaptations. Religious practices, such as novenas and community prayers, complemented institutional responses. The study underscores the role of colonial governance in adapting under epidemic pressures, illustrating an emergent institutional resilience. Conclusions: The 1742–1743 plague along the Royal Road serves as a case study for understanding the intersection of health crises and institutional adaptability. The review emphasizes the importance of coordinated public health measures and governance in addressing pandemics, offering lessons on resilience and social reconstruction applicable to contemporary health crises. This historical perspective enriches current discussions on crisis management and public health policy.

## 1. Introduction

The 18th century in South America was marked by profound socio-political and economic transformations as European colonization intensified, and Bourbon reforms sought to centralize governance and strengthen the Crown’s authority [1]. The restructuring of political, administrative, and fiscal systems sought to centralize authority, limit the power of local officials, and increase tax revenues. This was achieved through the establishment of new viceroyalties, such as the Viceroyalty of the Río de la Plata, and the imposition of trade monopolies on goods like tobacco and spirits. While these reforms succeeded in streamlining colonial governance, they also generated considerable social tensions, which later contributed to the independence movements of the 19th century [2].

**Figure 1:**
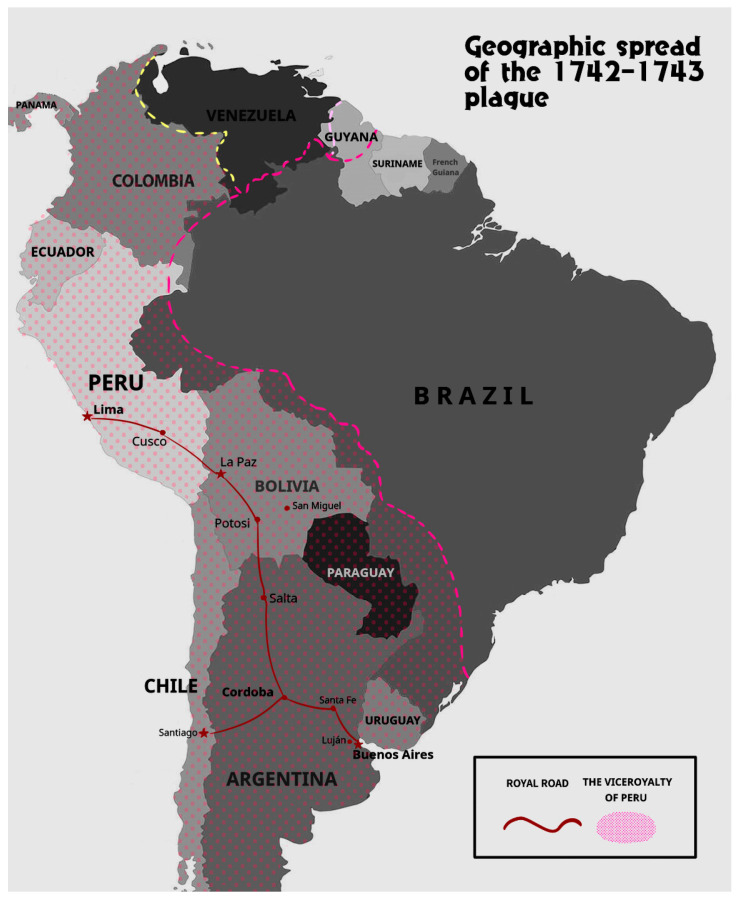
Geographic spread of the 1742–1743 plague along the Royal Road between Buenos Aires and Lima.

Amid these structural changes, South American societies faced recurring health crises, with epidemics posing substantial challenges to colonial governance and testing local resilience. The plague epidemic of 1742–1743, in particular, presented a significant threat, spreading through multiple cities along the Royal Road (Camino Real), which connected Buenos Aires and Lima, and resulting in widespread demographic impact. The Royal Road, a critical trade and communication route, facilitated not only economic exchange but also, inadvertently, the spread of the epidemic as people and goods moved quickly between major cities and towns along this commercial corridor [3]. Before the 1742–1743 epidemic, South America had already experienced significant outbreaks, such as the large epidemic that ravaged the southern Andes between 1714 and 1720, reaching its peak in Cusco in 1720, where thousands perished. Although this “peste grande” is not conclusively attributed to Yersinia pestis, it highlights the region’s endemic vulnerability to epidemics [4,5]. Similarly, historical records reveal that mortality rates rose across various sites along the Royal Road in the period leading up to and during the 1742–1743 epidemic. For example, records from Luján (Buenos Aires) document an increase in deaths beginning in late 1741, which aligns with data from Santa Fe [6]. Additionally, Jesuit records from the Chiquitos missions note an outbreak in San Miguel (Bolivia) at the end of 1741, suggesting a pattern of epidemic spread across multiple interconnected locations along the Royal Road [7,8]. In Córdoba, the epidemic is characterized as one of the region’s most severe crises, as documented by a marked increase in burials recorded in the Cathedral’s registers [9]. Bishop José de Peralta further emphasized the demographic toll in 1743, reporting that his diocese’s population would have nearly doubled if not for the significant mortality attributed to the “pestilence” [6] (Figure 1).

Institutional responses to this health crisis reveal the importance of religious practices and early public health interventions within colonial cities. A pertinent example is the city council of Santa Fe, Argentina, which, on 11 November 1741, authorized a novena of masses dedicated to Saint Jerónimo in supplication for relief from the epidemic and drought afflicting the region [10]. Shortly after, on 9 January 1742, the council again responded to the repercussions of the ongoing epidemic by ordering a novena to Saint Roque, the saint invoked for protection against plagues, underscoring the community’s desperation and hope for divine intercession in relief efforts [11]. In Córdoba, similar religious responses were paired with administrative recognition of the epidemic’s demographic impact. Such institutional accounts support the broader response strategies observed along the Royal Road, including cities such as Santa Fe and Luján, and Jesuit missions like San Miguel in Bolivia [8]. These communities combined religious ceremonies with preventive strategies, such as quarantines and isolation protocols, to mitigate the epidemic’s spread and address rising mortality rates.

Resilience is a central concept for understanding how these societies managed to confront and recover from severe epidemic challenges. In this context, resilience refers to the capacity of institutions and communities to absorb public health shocks, adapt to emergent conditions, and initiate post-crisis reconstruction. The Royal Road played an essential role not only in disseminating the epidemic but also in shaping the responses it provoked. Colonial authorities along this route implemented various public health measures, including quarantines, isolation protocols, and administrative reforms, aiming both to contain the epidemic and to mitigate its long-term effects. Despite the prevailing miasmatic theories of disease transmission, these early administrative efforts represent formative crisis management approaches that laid the groundwork for subsequent public health policies. Historical evidence and recent studies, such as the work by Frías and Montserrat [6], indicate that similar epidemics sequentially affected urban centers along the Royal Road, including Buenos Aires and Lima, supporting the hypothesis of a sequential spread of disease and highlighting the interconnectedness of urban centers in colonial health crises.

This study aims to explore how the plague epidemic along the Royal Road not only tested the colonial administrative apparatus but also catalyzed processes of social resilience and reconstruction. The resilience strategies implemented in response to the 1742–1743 plague demonstrate the ability of colonial authorities to adapt governance structures to manage widespread public health emergencies. By examining how these authorities adjusted their political and social frameworks during the crisis, this paper provides insights into how epidemics influenced both immediate responses and the long-term evolution of colonial institutions. Ultimately, this analysis seeks to shed light on the role of resilience in recovery and reconstruction following significant health crises in 18th-century South America. The understanding of these dynamics offers valuable lessons for contemporary public health crisis management, especially concerning how societies and governments can navigate the intersection of health, governance, and resilience in the face of pandemics.

## 2. Methods

This narrative review analyzes the historical and social responses to the 1742–1743 plague along the Royal Road, focusing on resilience strategies and social reconstruction. A systematic search of primary and secondary historical sources was conducted to identify public health measures and administrative actions taken by colonial authorities during the epidemic.

Sources were gathered from key historical archives, including the Archivo General de Indias, Archivo General de la Nación Argentina, and the Biblioteca Nacional de España. Additionally, peer-reviewed articles were accessed through databases such as JSTOR, Scopus, and Google Scholar. Search terms included “1742 plague”, “Camino Real”, “colonial resilience”, “epidemic responses”, “social reconstruction”, “quarantine”, “colonial public health”, and “*Yersinia pestis*”.

Relevant sources discussing the 1742–1743 plague, colonial administrative responses, and the social and economic impacts of the epidemic were selected. Priority was given to studies focusing on resilience strategies and public health interventions. Sources were reviewed for their relevance, and any discrepancies were resolved through discussion.

Key data on public health measures—such as quarantines, hospital construction, and governance reforms—were extracted and analyzed within the framework of resilience theory. The review also examined post-epidemic social reconstruction efforts and long-term institutional changes.

## 3. Results

In the 18th-century South American context, resilience was a transformative force that enabled societies to face and overcome adversities such as European conquest and widespread epidemics. This resilience went beyond mere survival, demonstrating a capacity to adapt, rebuild, and thrive in challenging conditions.

### 3.1. Importance of Resilience and Post-Epidemic Reconstruction Efforts

Resilience in 18th-century South America embodied more than survival; it facilitated adaptation and transformation in response to severe challenges. Scholars like Oriol (2012) [12] and García (2020) [13] have emphasized resilience’s role in overcoming past tragedies, suggesting that the capacity to acknowledge and learn from history fosters the ability to confront and transcend challenges such as genocides and marginalization.

### 3.2. Analysis of Individual and Collective Resilience in the Face of Adversity

Ruiz (2001) [14] examined the plague caused by Yersinia pestis, highlighting how past epidemics significantly impacted and altered social structures and community dynamics while also illustrating the role of environmental factors such as rodent populations and their ecological conditions in facilitating outbreaks. Moreover, as demonstrated by Frías and Montserrat [6], epidemics in the Río de la Plata and Tucumán regions drove the development of resilience strategies. These strategies not only allowed populations to survive but also promoted long-term social and cultural adaptation. Their work emphasizes that resilience was often spurred by the economic and social shifts that these health crises necessitated, as communities were forced to reconfigure their resource distribution and interpersonal connections to manage recurring threats.

From a colonial perspective, these cases of resilience can be interpreted as testimonies of overcoming and adapting under the colonial system. The policies implemented during epidemics could be presented as crucial for controlling and overcoming these health challenges, demonstrating the effectiveness and responsibility of colonial authorities in crisis management.

To provide a clearer understanding of the timeline and geographic spread of key epidemic events, Table 1 summarizes the major outbreaks and the corresponding public health responses in 18th-century South America.

### 3.3. The Influence of Global Perspectives on Resilience Practices

Resilience strategies adopted in response to epidemics and conquests illustrate how colonial authorities indirectly contributed to the transformation of social practices [6]. Although emerging in a context of oppression, these practices can be seen as reflections of colonial oversight that allowed for the reconfiguration and growth of new forms of organization and resistance. Resilience thus becomes a lens through which the history of conquest and epidemics in South America is reevaluated, highlighting adaptation, resistance, and transformation under colonial influence.

This interpretation provides a perspective that values the complexity of human resilience and administrative capacities in historical contexts marked by forced and often violent changes, offering valuable lessons on crisis management and cultural survival under colonial rule.

The examination of the 18th-century South American context reveals that epidemics had a profound impact on the demographic dynamics and social cohesion of communities. For example, while Mexico City [15] benefitted from early centralized health measures under the Spanish Crown (beginning in the 1720s), including the establishment of hospitals and hygiene regulations coordinated through the Protomedicato Tribunal, South American colonies often lacked the same level of systematic public health responses. This disparity highlights the fragmented and decentralized nature of health interventions in South America, which, though flexible, were often less effective in mitigating the full impacts of epidemics.

In 1741, the city council of Santa Fe in Argentina ordered a novena of masses in response to the combined threat of plague and drought, reflecting the role of religious practices in fostering social cohesion during health crises. The same council, on 9 January, initiated another novena to Saint Roque in hopes of divine intervention during the ongoing epidemic, underscoring the reliance on spiritual responses in times of crisis [10,11]. Simultaneously, Lima [16] faced multiple epidemics that devastated its population, prompting significant public health measures. Faced with the recurrence of these health crises, local authorities were compelled to establish public health institutions and develop policies to control and prevent future outbreaks. These initiatives represent an emerging effort by authorities to tackle significant public health challenges. The resulting high mortality drastically altered the city’s demographic composition, significantly impacting labor and economic dynamics and, consequently, social development and community structure. Additionally, this juncture forced Lima society to adapt its cultural and religious practices; rituals and festivals transformed to incorporate elements of supplication and divine protection against diseases, demonstrating a profound interaction between cultural beliefs and responses to health emergencies.

The outbreak of matlazahuatl (1762–1763) in the Jurisdiction of Villa de Córdoba provides another example of how environmental factors influenced epidemic spread. Sánchez et al. (2016) documented how some towns requested tax relief due to the epidemic’s impact while others were spared, possibly due to climatic differences. The case illustrates how local environmental conditions played a critical role in shaping the response to health crises [17].

The hospital structure in Lima during the viceregal period offers another significant example of the response to health crises in the region. Villamón (2022) [18] describes how hospital architecture reflected a new awareness for addressing health issues and imbuing them with a religious character. Hospitals in Lima were built with a cross-shaped plan typology, allowing better organization and separation of patients and favoring ventilation and natural light. This cruciform arrangement was functional for light and ventilation, as the sickrooms were spacious and long, with religious services located in the center of the transept, thus concentrating the patients’ spiritual and material needs [18].

These cases demonstrate the decentralized and often fragmented nature of resilience strategies in South America, which, though flexible, were less cohesive compared to centralized responses in regions like Mexico City.

## 4. Discussion

In 18th-century South America, epidemics represented not only significant health crises but also posed critical challenges to colonial power structures. The Bourbon reforms, aimed at centralizing and strengthening colonial control, had a profound impact on the management of these crises in both Europe and the Americas. As Zarzoso (1999) [19] highlights, during the epidemics in Catalonia, Bourbon authorities implemented a range of public health measures, including the construction of hospitals, the provision of food and medicine, and broader administrative reforms to ensure the continuity of economic production and social order. These efforts were mirrored in South America during the 1742–1743 plague along the Royal Road between Buenos Aires and Lima, where colonial authorities introduced similar strategies, such as quarantines, the establishment of temporary hospitals, and tax suspensions, all aimed at maintaining both public health and economic stability. Through a detailed historical analysis of primary and secondary sources, this study documents these responses, offering a comprehensive timeline of the epidemic and highlighting how colonial institutions adapted to mitigate its impacts. These strategies of resilience and social reconstruction underscore the capacity of colonial governance to evolve in the face of adversity, providing valuable insights into crisis management and emphasizing the importance of robust health systems and adaptive governance for addressing future global crises.

### 4.1. Social Reconstruction Post-Epidemics

The resilience and social and economic reconstruction following health crises in the 18th century in South America bear testimony to the human capacity to confront and overcome moments of great adversity. The response to these crises was multifaceted, involving both colonial authorities and the Church and prompting significant changes in social structures and cultural adaptations. Our findings emphasize the importance of resilience and social reconstruction in communities affected by the epidemic along the Royal Road between Buenos Aires and Lima. Notably, while historical sources present limitations in identifying the exact pathogen responsible for the epidemic, this ambiguity does not detract from the primary focus of our study, which centers on examining the epidemic’s social and demographic effects. This focus on social resilience offers a valuable perspective on community responses and adaptations during the crisis, irrespective of the specific etiology. These results highlight the importance of investigating social responses to health crises, revealing adaptive processes that contributed to the continuity and reorganization of social structures in that era.

### 4.2. Social and Economic Reconstruction Strategies

After the epidemics, social and economic reconstruction strategies varied according to regions and specific circumstances. However, common efforts were directed toward population recovery, infrastructure rebuilding, economic revitalization, and the restoration of public health systems. Authorities implemented policies to encourage the repopulation and reconstruction of devastated areas, including incentives for immigration, land redistribution, and support for local agriculture and production.

### 4.3. The Role of Colonial Authorities and the Church

During the colonial period, both governmental authorities and the Catholic Church played pivotal roles in the coordination of epidemic responses, with efforts centered on mitigating demographic and economic disruptions through legislative interventions, resource management, and spiritual support. Acting within the centralized framework imposed by the Bourbon monarchy, colonial authorities implemented critical public health measures, including quarantines and the construction of hospitals, to safeguard public health and ensure economic continuity. Simultaneously, the Catholic Church contributed to both healthcare and spiritual well-being by establishing hospitals and leading religious practices, such as novenas, aimed at seeking divine intervention during crises. In South America, these religious practices exhibited a unique syncretism, blending indigenous traditions with Catholic rituals, which bolstered the cultural resilience of affected communities [19]. However, the Church’s capacity to provide medical care varied geographically, being more effective in areas such as Mexico City [15], where ecclesiastical infrastructure was more advanced, but less effective in other parts of South America due to limited resources.

Moreover, cultural and political repertoires significantly shaped the management of epidemics throughout the Spanish Empire, with marked differences between responses in Europe and in the colonies. In South America, the presence of indigenous populations and African slaves introduced additional complexities to public health interventions [20]. Indigenous knowledge systems, while insufficient to fully address the newly introduced diseases, provided a foundation for localized resistance and recovery efforts, distinct from European approaches. The political objectives of the Bourbon reforms, which emphasized consolidating colonial control and economic stability, often conflicted with the needs of local populations, as the well-being of indigenous and enslaved communities was frequently subordinated to imperial interests [21]. These tensions, coupled with the centralized decision-making processes dictated by the Crown, heavily influenced the colonial response to public health crises.

### 4.4. Changes in Social Structures and Cultural Adaptations

Epidemics led to significant shifts in social structures and cultural adaptations. The reduction in indigenous populations increased reliance on African slave labor, leading to demographic and cultural transformations. This blend of indigenous, African, and European influences contributed to the region’s evolving cultural identity. The high mortality rates also impacted labor and economic dynamics, driving changes in community structures and practices, including the transformation of religious rituals to incorporate supplication and divine protection against diseases.

In Córdoba, individual responses to epidemics also involved legal strategies to mitigate the socioeconomic challenges posed by health crises. For example, during a severe outbreak in 1742, Doña Bartolina Cabrera, a member of a prominent local lineage, employed legal mechanisms to ensure her family’s economic and hereditary stability by delegating the management of her estate to a trusted relative through a will [22]. An illustrative example of collective adaptation is documented during the matlazahuatl outbreak (1762–1763) in the towns of the Jurisdiction of Villa de Córdoba. Sánchez et al. (2016) [21] documented that the devastation led local populations to request tax relief, reflecting an administrative response to economic hardship. Interestingly, some population centers, such as San Jerónimo Zentla, San Juan de la Punta, and Santiago Huatusco, were spared, possibly due to their warmer climates. Testimonies and historical reports suggest that climatic conditions played a critical role in the uneven spread of the disease.

Another significant response to health crises in the region was seen in Lima’s hospital infrastructure during the viceregal period. Villamón (2022) [18] describes how hospital architecture reflected a new awareness of addressing health issues, imbuing them with a religious character. Hospitals in Lima, built with a cross-shaped floor plan typology, allowed for better organization and separation of patients, promoting ventilation and natural light. This cruciform layout was functional for light and ventilation, as the sick rooms were spacious and long, with religious services located in the center of the crossing, thus concentrating the spiritual and material needs of the patients.

Resilience in the context of 18th-century epidemics in South America was not just a matter of survival but also of adaptation and transformation. The capacity of societies to rebuild after health crises highlights the importance of solidarity, cooperation, and innovation in recovery processes. The lessons learned from these historical experiences are fundamental for understanding the complexity of resilience and social reconstruction in times of crisis, offering valuable perspectives for facing contemporary and future challenges.

Additionally, it is important to note that responses to epidemics in 18th-century South America were influenced not only by social and cultural factors but also by colonial political structures. In regions like northern Argentina, decentralization and ongoing negotiations with local leaders were key in managing territories and implementing crisis response strategies. This lack of centralized political control contrasts with the more systematic and effective public health policies implemented by the Spanish Crown in cities like Mexico, where coordinated governance allowed for more resilient communities in the face of epidemics [23].

## 5. Conclusions

This study provides a detailed examination of the resilience and social reconstruction strategies implemented by colonial authorities in response to the 1742–1743 plague along the Royal Road between Buenos Aires and Lima. The findings highlight how the application of quarantine measures, the construction of temporary hospitals, and administrative reforms were critical in mitigating the epidemic’s immediate and long-term impacts. These measures, adapted from the Bourbon reforms in Spain, showcase the flexibility of colonial governance under extreme public health pressures.

The study also reveals the nuanced role of the Catholic Church in providing both medical and spiritual support, which, combined with the centralized political responses, contributed to shaping post-epidemic recovery efforts. The social and economic reconstruction strategies, including tax relief and land redistribution, were vital in restoring the region’s demographic and economic stability.

The adaptive strategies observed in this historical case provide valuable insights into contemporary crisis management. The successful combination of public health measures and governance adaptation demonstrates the importance of cohesive institutional responses to health crises. Moreover, this study highlights the crucial role of resilience in ensuring the recovery and continuity of social and economic systems in the face of epidemics, a lesson that remains highly relevant in the management of modern global health challenges.

## Figures and Tables

**Table 1:** Timeline of key epidemic events and responses in 18th-century South America.

Year	Event/Epidemic	Location	Key Responses
1741	Plague and Drought in Santa Fe	Santa Fe, Argentina	Novena masses ordered in supplication to Patron Saint San Jerónimo due to the plague and drought.
1742–1743	Plague along the Royal Road	Camino Real (Buenos Aires to Lima)	Sequential spread along the Royal Road, with rising mortality in late 1741 in Luján and Córdoba. Regional measures included quarantines, public health actions, and a novena to Saint Roque in Santa Fe. Peak mortality in Buenos Aires (128 deaths in June 1742) and 160 fatalities at Purísima Concepción de los Pampas. Jesuit records confirm an outbreak in San Miguel, Bolivia.
1762–1763	Matlazahuatl Epidemic	Jurisdiction of Villa de Córdoba, Mexico	Requests for tax relief; warmer climate may have spared certain towns (San Jerónimo Zentla, San Juan de la Punta, Santiago Huatusco).
Mid-18th century	Epidemics in Lima	Lima, Peru	Establishment of public health measures, construction of hospitals with cross-shaped architecture.
1780s	Various Epidemics	Lima, Peru	Health crises prompted further public health interventions and adjustments in religious and cultural practices.
